# Photochemical Degradation of Cyanides and Thiocyanates from an Industrial Wastewater

**DOI:** 10.3390/molecules24071373

**Published:** 2019-04-08

**Authors:** Juan Jose Viña Mediavilla, Begoña Fernandez Perez, Maria C. Fernandez de Cordoba, Julia Ayala Espina, Conchi O. Ania

**Affiliations:** 1 Escuela Técnica Superior de Ingeniería de Minas, Universidad de Oviedo, 33001 Oviedo, Spain; UO45242@uniovi.es (J.J.V.M.); jayala@uniovi.es (J.A.E.); 2 CEMHTI, CNRS (UPR 3079), Université d’Orléans, 45071 Orléans, France; maria.fdecordoba@cnrs-orleans.fr; 3 Instituto Nacional del Carbón (INCAR, CSIC), Apdo. 73, 33080 Oviedo, Spain

**Keywords:** cyanides, thiocyanates, oxidation, photooxidation, solar light

## Abstract

We have explored the simultaneous degradation of cyanides and thiocyanate present in wastewaters from a cokemaking factory using photoassisted methods under varied illumination conditions (from simulated solar light to UV light). Overall, the photochemical degradation of cyanides was more efficient than that of thiocyanates, regardless of the illumination conditions, the effect being more pronounced in the absence of a photocatalyst. This is due to their different degradation mechanism that in the case of thiocyanates is dominated by fast recombination reactions and/or charge transfer reactions to electron scavengers. In all cases, cyanate, ammonia, nitrates, and nitrites were formed at different amounts depending on the illumination conditions. The conversion yield under simulated solar light was almost complete for cyanides and quite high for thiocyanates after 6 h of illumination. Regarding toxicity, photochemical oxidation at 254 nm and under simulated solar light decreased significantly the toxicity of the pristine wastewater, showing a correlation with the intensity of the irradiation source. This indicate that simulated light can be effectively used to reduce the toxicity of industrial effluents, opening an interesting perspective for optimizing cyanide detoxification systems based on natural light.

## 1. Introduction

Cyanides and thiocyanates are formed in several industrial processes such as gold mining activities and the production of coke in steel factories. Both pose major environmental and health concerns as they can be generated in high concentrations in industrial effluents [1,2]. For instance, typical consumptions of cyanide in cyanidation plants are ca. 100 times higher than the stoichiometric amounts due to the large losses associated to the formation of volatile HCN, cyanide metallic complexes (copper, iron), and thiocyanates. Thus, the concentrations of cyanides and thiocyanates in mill effluents in gold mining activities can vary considerably between 40 to 600 mg/L [3]. In cokemaking factories, thiocyanate is one of the major constituents of wastewater with concentrations in the range of 20–1500 mg/L. Thiocyanate can also be formed biologically from the detoxification of cyanides [3]. Furthermore, although thiocyanate is less toxic than cyanide, it is more stable and thus more difficult to degrade.

The co-existence of thiocyanate and cyanides in such effluents can result in the contamination of the receiving water bodies. In order to meet regulatory discharge standards, it is important to develop processes to reduce the concentration of both species down to acceptable levels before discharge. To attain this goal, efforts focus mainly in either promoting recovery and reuse approaches in the industry or applying degradation techniques to decompose both cyanide species in harmless compounds [4,5]. Various chemical and biological technologies have been proposed for the destruction of cyanides [4,6,7,8,9,10]. Among them, biodegradation treatments (such as activated sludge, biofilms, bacterial co-culture) have gained popularity due to their robustness, high efficiency in batch systems, and economic viability. However, the scaling up of this technology from laboratory to full scale still represents a major challenge and few studies have focused on the simultaneous biological degradation of cyanides and thiocyanates, with little knowledge on the bacterial co-metabolism of these contaminants in continuous systems [5]. 

On the other hand, various oxidation processes have been investigated as alternatives for the degradation of cyanides and thiocyanates from industrial and mining effluents [6,7,9,11]. The use of oxidizing agents (e.g., ozone, hydrogen peroxide, SO_2_/air) has become very popular. Despite their effectiveness, these are quite expensive approaches that still lead to the production of hazardous by- or end-products in the treated effluent. On the other hand, the use of photoassisted techniques for the degradation of cyanides seems an interesting alternative [12,13,14,15], while it has not been much explored for the degradation of thiocyanates [16,17,18]. 

Taking this into account, the objective of this work was to investigate the simultaneous degradation of cyanides and thiocyanates typically present in industrial wastewaters using a photochemical approach. We explored different irradiation conditions from simulated solar light to UV light, as well as the use of a photocatalyst (e.g., commercial TiO_2_) to compare the efficiency of the photochemical approaches applied to both synthetic and wastewaters from a cokemaking factory containing cyanides and thiocyanates. Special attention has been paid to the different degradation intermediates generated in the studied conditions and to the toxicity of the solutions after the photochemical treatment. 

## 2. Materials and Methods

### 2.1. Chemicals

Potassium cyanide, potassium thiocyanate, chloramine-T, isonicotinic acid and 1,3-diethyl barbituric acid, potassium nitrate, potassium nitrite, ammonium chloride, potassium cyanate, potassium sulfate were purchased from Sigma-Aldrich (Darmstadt, Germany). All chemicals and standard solutions were of analytical reagent grade (Sigma-Aldrich) and all aqueous solutions were prepared ultrapure water (Millipore Milli-Q system, Darmstadt, Germany).

### 2.2. Industrial Wastewater

The composition of the industrial wastewater from a cokemaking factory used for the photodegradation studies is summarized in Table 1 through the main physiochemical parameters. 

### 2.3. Light Sources

The photodegradation experiments were performed using various irradiation sources with different power and wavelengths aiming to evaluate their performance and applicability in the photodegradation of cyanide species: (i) simulated solar light was provided by a 300 W Osram Ultra-Vitalux lamp (Munich, Germany) and will be labeled as SimSolar; (ii) a high-pressure mercury UV lamp (Helios Italquatz, Milan, Italy) emitting at 254 nm (UVC lamp); (iii) a low pressure UV-Vis lamp (Helios Italquatz) emitting between 200–600 nm (UV-Vis lamp). Further details on the different illumination sources are summarized in Table 2. The irradiance of each lamp was measured using a photodiode (Thorlabs, Milan, Italy), and their corresponding emission spectra were recorded in a fluorometer and are shown in Figure S1 in the Supporting Information.

Italy
db Electronic Instruments srl
Via Teano, 2
20161 Milano
Italy
[Photonics]
Sales:
P: +39-02646-9341
F: +39-02645-6632
E: sales@dblaser.it
Support:

### 2.4. Photochemical and Photocatalytic Degradation Experiments

The photodegradation experiments were performed at room temperature in a 500 mL batch reactor with cyanide and thiocyanate solutions of different initial concentration. Before illumination, the suspensions were stirred in the dark for 20 min. An excess of oxygen during the photocatalytic runs was supplied by a continuous air flow. Aliquots of the solution were removed periodically during the photocatalytic assays experiments and filtered (0.45 mm nylon filter) before measuring the concentration of cyanide species and their degradation intermediates. Bubblers filled with sulfuric acid and sodium hydroxide solutions were installed at the end of the photoreactor to trap any loss of ammonia and hydrocyanic acid during the photocatalytic experiments (due to the continuous air supply). Photodegradation experiments were performed in the absence and presence of a photocatalyst; for the latter, commercial TiO_2_ powders (P25, Evonik, Essen, Germany) were used as benchmark catalyst, with a catalyst loading of 250 mg/L. The main characteristics of the semiconductor are compiled in Table S1. All photodegradation experiments were performed at least in duplicate; average data is herein presented (accuracy of ca. 5%). 

### 2.5. Analytical Methods

Total cyanides were measured using a flow injection analysis (FIA) method (ISO 14403-1) [19] at pH of 6–8 with colorimetric detection (chloramine-T, isonicotinic acid and 1,3-diethyl barbituric acid) immediately after taking samples [20]. The resulting reaction product is a blue dye that can be detected at 590–610 nm. The system (Seal Analytical Ltd, Southampton, United Kingdom) is provided with a UV source in the digestion module that allows the cleavage of any metal-cyanide complexes, resulting in the release of bound cyanides. Thiocyanates do not interfere in the determination of cyanides using this FIA method. The concentrations of thiocyanate (SCN^−^), as well as intermediate degradation ions such as sulfate (SO_4_^2−^), cyanate (CNO^−^), nitrite (NO_2_^−^), nitrate (NO_3_^−^), and ammonium (NH_4_^+^) were monitored in an ion exchange liquid chromatograph (Metrohm 850 IC, Herisau, Switzerland) with a suppressed conductivity detector and operating at 40 °C. For the determination of the cationic species (column Metrosep C3 250/4.0, 4 mm × 250 mm, 5 µm) a solution of 3.5 mM HNO_3_ was used as eluent (flow rate 0.5 mL/min). For the anionic species (column Metrosep A Supp 7-250, 4 mm × 250 mm, 5 µm), the eluent was 3.6 mM Na_2_CO_3_ (flow rate 0.5 mL/min). The injection volume was 20 µL using samples previously filtered (0.45 µm nylon filter). All analytical measurements were made at least in triplicate, with a standard deviation below 5% in all cases. 

### 2.6. Toxicity Measurements

The toxicity of the solutions was evaluated by the luminescence inhibition of the marine bacterium Vibrio fischeri test, according to international procedures (ISO 11348-3) [21]. The tests were carried out in a Microtox M500 Analyzer (Modern Water, Surrey, United Kingdom) according to the Microtox Manual (1992: standard procedure) for the phenol controls. The bioluminescent marine bacteria were provided by Hach Lange France SAS (Lognes, France). The luminescent bacteria were exposed to dilutions in the range of 50–5% of the solutions previously filtered (0.45 micron nylon filter) in Microtox Diluent (Surrey, United Kingdom) (2% NaCl solution) at 15 °C. Osmotic adjustment of the solutions was carried out in 22 wt.% NaCl; the pH was adjusted to 6–8 when necessary. The light intensity of the samples was recorded after 5 and 15 min; results after 15 min are reported. The sensitivity of the bacteria was previously tested using reference compounds (phenol and zinc sulphate). For the synthetic solutions, the mean effective concentration parameter (EC50) was calculated. All the tests were performed at least in duplicate for all the dilutions. 

## 3. Results and Discussion

### 3.1. Characteristics of Light Sources

Table 2 summarizes the characteristics of the different light sources used in terms of output wavelength range and irradiance. The corresponding spectra are shown in Figure S1. Besides the differences in the output power, the selected lamps displayed important differences in the emission spectra (Figure S1). UVC and UV-Vis lamps emit at specific wavelengths, with one spectral line at 254 nm and several lines in the range of 200–600 nm, respectively. In contrast, the simulated solar light provided by the Xe lamp displayed a continuum emission in the range of 200–800 nm centered at about 600 nm and with a low contribution at wavelengths below 400 nm (mimicking sunlight). This allowed exploration of the impact of the high and low energy photons in the degradation reaction. 

### 3.2. Photochemical Degradation

**Figure 1:**
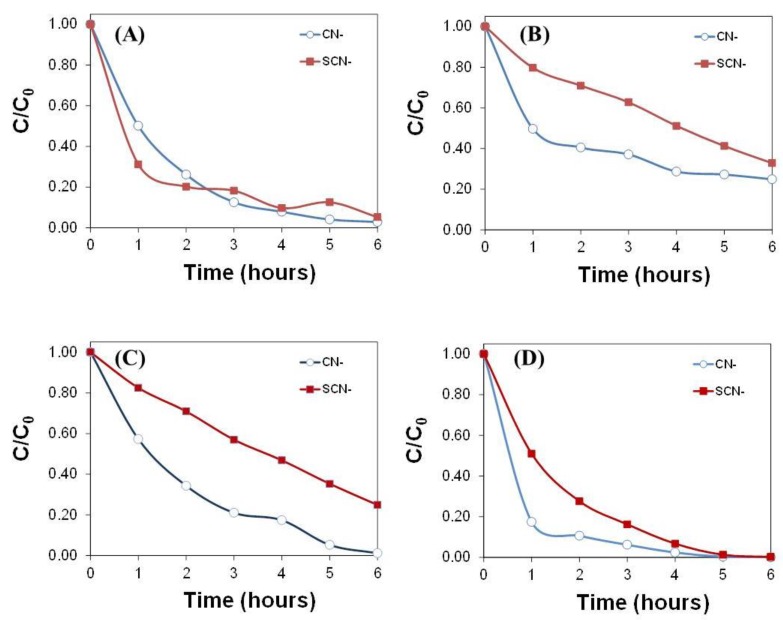
Evolution of the concentration of cyanides and thiocyanates in the wastewater upon illumination with various light sources: (**A**) UVC lamp; (**B**) UV-Vis lamp; (**C**) SimSolar lamp; (**D**) SimSolar lamp in the presence of TiO_2_ as photocatalyst.

**Figure 2:**
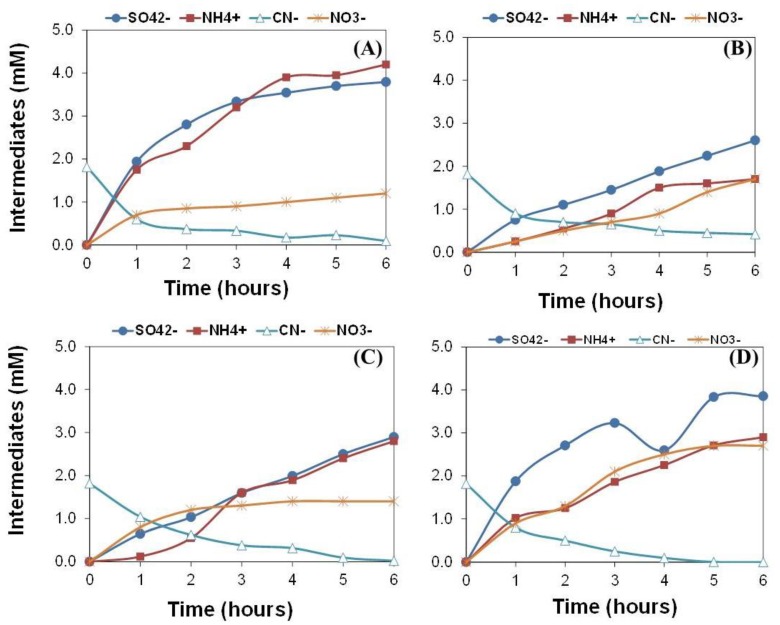
Intermediates detected in the wastewater upon illumination with various light sources: (**A**) UVC lamp; (**B**) UV-Vis lamp; (**C**) SimSolar lamp; (**D**) SimSolar lamp in the presence of TiO_2_ as photocatalyst.

Several sets of experiments were conducted to compare the photooxidation performance of cyanide and thiocyanates in the absence and presence of titania powder as photocatalyst. The evolution of the concentration of both cyanide species over time (as well as the intermediates formed) was monitored for the different light sources, and results are given in Figure 1 and Figure 2. For all the illumination conditions, the degradation yield of cyanides after 6 h of irradiation was significantly higher than that of thiocyanates. Differences are also noticed regarding the disappearance rate, with over 50% of cyanide conversion being achieved after 2 h of illumination for all the lamps, compared to 3 to 5 h needed to achieve the same conversion in the case of thiocyanates. This indicates that not only are thiocyanates more stable (thus less degraded) than cyanides, but the conversion rate is also slower. The poor conversion of thiocyanates has also been reported for several oxidation processes (chemical and photochemical) [7,8,22]. 

Regarding the illumination conditions, the conversion of cyanides followed the trend: UVC lamp > SimSolar > UV-Vis lamp, whereas for thiocyanates the degradation yield achieved with the simulated solar light and the UV-Vis lamp was quite similar. For both cyanide species, the best performance was obtained with a UVC lamp, with degradation efficiencies close to 100% after 4 h of illumination. This is interesting considering that this lamp displayed the lowest output power (Table 2) of the studied illumination sources. It also indicates that the monochromatic character of this lamp with a specific emission wavelength centered at 254 nm is more adapted than the polychromatic spectra of UV-Vis and SimSolar that emit in a wider spectral range. Thus, the average output power is shared by all the spectral range emitted by the lamp. Such differences in the performance were more pronounced in the case of thiocyanates, pointing out that the contribution of light above 254 nm (less energetic) is less effective to degrade this molecule. Similar observations have been reported for the oxidation of cyanides with lamps emitting above 300 nm [11]. 

Furthermore, under simulated solar light, the degradation of cyanides is almost complete after 6 h of illumination, and the conversion of thiocyanates is about 70%. This is most outstanding considering that it is achieved with sunlight illumination conditions in the absence of a catalyst and demonstrates the feasibility of degradation systems based on natural illumination conditions (e.g., ponds) to simultaneously degrade both cyanide species (although optimization of such a system would still be needed). Under simulated solar light, the degradation experiments were also carried out in the presence of commercial titania as photocatalyst (ca. loading of 250 mg/L). Data showed a large increase in the conversion of both cyanide species in the presence of the photocatalyst, achieving the complete degradation of both species after 5 h of illumination. The influence of TiO_2_ is was more remarkable during the first couple of hours of irradiation. An increased conversion in the degradation of cyanides in the presence of titania has been reported in the literature for irradiation mainly using UV light [23,24]. We herein observe an important increase in the performance using sunlight, which is interesting given that this semiconductor is characterized by a low efficiency under solar light due to its optical features (Table S1). Also, it is important to mention that the adsorption of cyanides and thiocyanates in TiO_2_ is negligible, in agreement with its textural features (Table S1).

**Figure 3:**
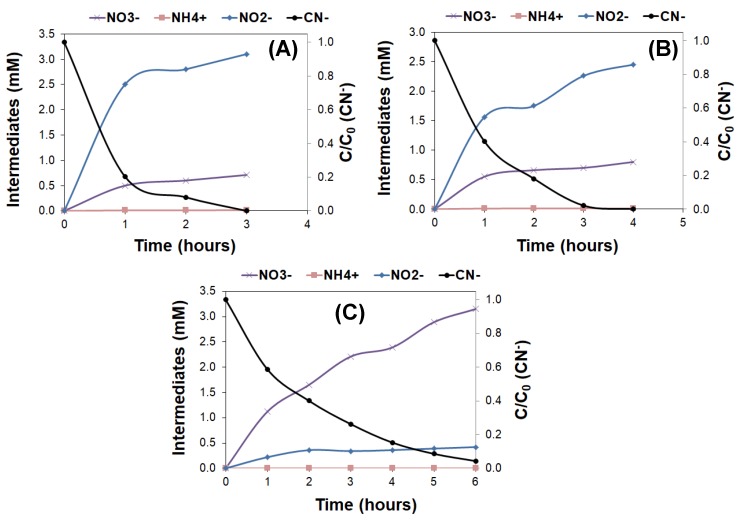
Evolution of the concentration of cyanides (left axis) and intermediates (right axis) upon illumination of a synthetic solution of 100 mg/L of KCN with various light sources. (**A**) UVC lamp; (**B**) UV-Vis lamp; (**C**) SimSolar lamp.

**Figure 4:**
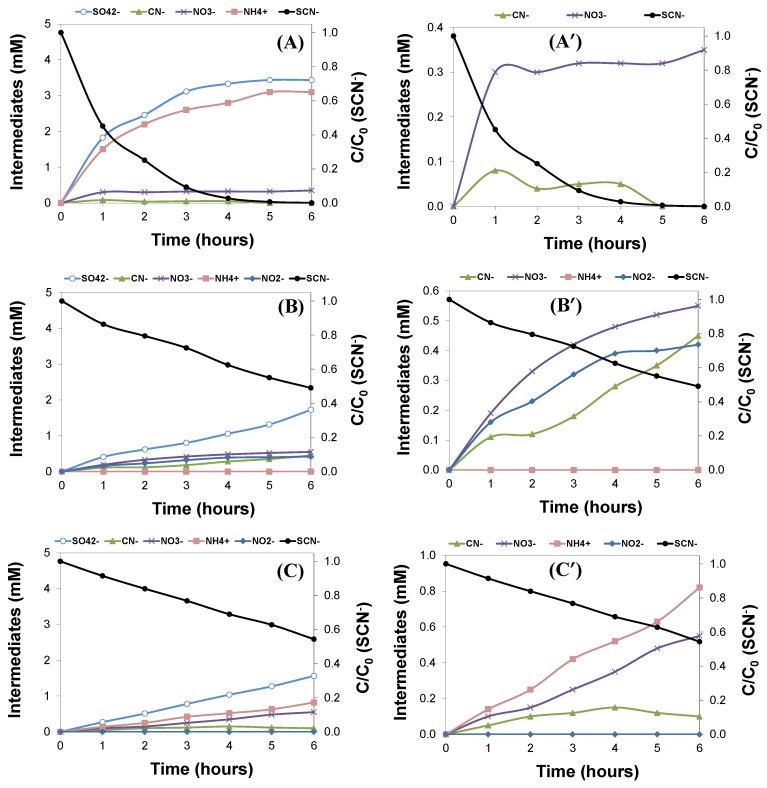
Evolution of the concentration of thiocyanates (right axis) and intermediates (left axis) upon illumination of a synthetic solution of 200 mg/L of potassium thiocyanate with various light sources. (**A**,**A’**) UVC lamp; (**B**,**B’**) UV-Vis lamp; (**C**,**C’**) SimSolar lamp (for clarity, plots A, B, and C are magnified in A’, B’, and C’, respectively).

Photocatalytic experiments of cyanide and thiocyanate synthetic solutions of similar initial concentrations (i.e., 100 and 200 mg/L, respectively) showed a similar degradation pattern under the different illumination conditions (Figure 3 and Figure 4). Indeed, the complete degradation of 200 mg/L of thiocyanates was achieved with a UVC lamp after 4–5 h, needing ca. 1 h less light exposure than the industrial wastewater to achieve full conversion.

The analysis of the intermediates confirmed the presence of nitrates, ammonium, and sulphates in the solution (Figure 2) with concentrations increasing continuously during the reaction time. Similar reaction pathways have been proposed in the literature for the photooxidation of cyanides, with slight differences in the speciation of intermediates. For instance, the absence of nitrites and nitrates has been reported and explained by an insufficient generation of oxidants in the photochemical processes [25]. The production of formamide, formic acid, isocyanic acid, and nitrous oxide has also been postulated in other chemical oxidation processes of thiocyanates [6,7], although none of them were detected under our experimental conditions. 

We have not detected the appearance of any solid deposit (e.g., sulfur) under our experimental conditions. This is important to assure homogeneous irradiation conditions during the photochemical assays as the presence of particles may filter a fraction of the incident radiation; they might also favor back-reactions with cyanides to regenerate thiocyanates. Additionally, cyanates were not detected in the solutions of the wastewater, although it has been reported that they are involved in the initial steps of the oxidation of cyanides [12,24]. In this case, we associate this to a fast hydrolysis of the cyanates formed, as has been reported for the chemical oxidation of cyanides in synthetic solutions in the early steps of the reactions [4,7]. 

Regarding the impact of the illumination source, higher concentrations of intermediates are formed upon irradiation at 254 nm, with similar trends for the other two lamps. Ammonium is clearly the predominant N-intermediate formed when the wastewater is exposed to the UVC lamp; the concentrations increase over time, reaching a somewhat steady state after 4 h of illumination. In contrast, when the wastewater is exposed to UV-Vis and SimSolar lamps, the concentration of nitrates reached values close to those of ammonium (although both at lower concentrations).

This contrast with the trend observed for the photochemical degradation of cyanides and thiocyanates from synthetic solutions with similar initial concentrations (Figure 4). Ammonium is the predominant N-intermediate in the photooxidation of thiocyanates from synthetic solutions, whereas nitrates and nitrites are the main N-intermediates detected in the photochemical oxidation of cyanides from a synthetic solution (regardless the irradiation source). 

Even though the conversion of thiocyanates was not high, the yield of thiocyanates to sulfate (Table 3) was closed to the maximum theoretical yield (ca. 1) for all the photochemical treatments. This demonstrates that sulfate is the main sulfur containing intermediate formed, as neither sulfides, disulfides, nor sulfur were identified during the reaction. The molar yields of thiocyanates and cyanides conversion to *N*- intermediates (nitrite, nitrate, and ammonium) were also quite high. The slight unbalance in this case suggests the formation of other *N*-containing intermediates (e.g., molecular nitrogen as suggested in the literature) [6] at certain stages of the reaction (Table 3). The analysis of the solution in the bubblers placed at the end of the gas line of the photochemical reactor showed that practically no HCN nor ammonia were released as gases, as the pH was kept constant during the reaction. As for carbon, it is most likely transformed to carbonate and bicarbonate (fractionation depends on the pH of the solution). 

### 3.3. Reaction Mechanism

A fundamental insight into the photochemical degradation of cyanide species in the presence and absence of a catalyst is crucial for further optimization and development of this approach. Taking into account our experimental data, the reaction pathway for the photochemical oxidation of cyanides and thiocyanates has been analyzed and is discussed below. It should be noted that the stoichiometry of the reactions has not been adjusted, since the discussed mechanism involves the plausible formation of transient radical species, which decomposition fate in a complex wastewater matrix cannot be unequivocally attributed. Nevertheless, final products are proposed considering the intermediates detected experimentally. 

According to literature and supported by herein gathered data, cyanide oxidation would be initiated by the light excitation and reaction with the photogenerated species such as pair electron/holes, hydroxyl, and superoxide radicals (r1)–(r7), leading to the formation of cyanate. This compound would be subsequently hydrolyzed (r8) or further oxidized (r9) into different final products such as ammonia, nitrates, nitrogen, or nitrites. Further photooxidation of ammonia to nitrates can proceed in basis medium, both in the absence and presence of a catalyst. The higher concentration of ammonium detected after exposure to a UVC lamp, compared to UV-Vis and SimSolar suggests that the rate of ammonia photoconversion depends on the illumination intensity. A similar trend has been reported for the photooxidation of ammonia solution with various lamps [26].

CN^−^ + hν → CN• → → (CN)_2_ (r1)

(CN)_2_ + HO^−^ → OCN^−^ + CN^−^ + H_2_O (r2)

H_2_O + hν → HO• (r3)

O_2_ + hν → O_2_• (r4)

HO• → H_2_O_2_ (r5)

CN^−^ + O_2_• → OCN^−^ (r6)

CN^−^ + 2OH• → OCN^−^ (r7)

OCN^−^ + H_2_O → CO_3_^2−^ + NH_3_ (ac) → NH_4_^+^ + OH^−^ (r8)

OCN^−^ + HO• → HCO_3_^−^ + N_2_ + H_2_O (r9)

NH_4_^+^ + hν + OH^−^ → NO_3_^−^/NO_2_^−^ + H_2_O (r10)

In parallel, the photochemical degradation of thiocyanates would be initiated by the formation of SCN• radical (r11) and thiocyanogen radical anion (r12) [17,27]. However, both species undergo fast recombination reactions, regenerating thiocyanates without yielding any net photochemical transformation (r13). This would account for the much lower conversions of thiocyanates compared to cyanides. In the presence of electron scavengers (such as oxygen or a photocatalyst surface) and of high intensity light sources (i.e., a UVC lamp), electrons are easily transferred, thus recombination is suppressed, and hence photochemical transformation reactions are favored (r14)–(r17).

SCN^−^ + hν → SCN• (r11)

SCN^−^ + SCN• → (SCN)_2_•^−^ (r12)

(SCN)_2_•^−^ → (SCN)_2_ + SCN^−^ (r13)

(SCN)_2_ + H_2_O → CN^−^ + SCN^−^ + SO_4_^2−^ + OH^−^ (r14)

SCN^−^ + hν → CN^−^ + S (r15)

S + SCN^−^ → CN^−^ + S_2_^2−^ (r16)

2S + 3O_2_ + 2H_2_O → 2SO_4_^2−^ + OH^−^ (r17)

Thiocyanate can also react with photogenerated O-radicals yielding hypothiocyanite (r18) and thiocyanogen (r19) [28]. Both species are rapidly hydrolyzed, leading to the formation of thiocyanate, cyanide, and sulfates (r14) [29].

SCN^−^ + 2HO• → OSCN^−^ + H_2_O (r18)

OSCN^−^ + SCN− + H_2_O → (SCN)_2_ + 2OH^−^ (r19)

In sum, even though photochemical approaches enable the breakdown of thiocyanates, in the absence of a catalyst to generate O-radicals, direct light activation is not enough to achieve complete oxidation (even after long illumination periods). This is due to the low light absorption coefficient of thiocyanates and its stability. A similar observation has been reported for various oxidizing agents (e.g., Inco SO_2_/air process [10] hydrogen peroxide [30]), typically leading to poor degradation efficiencies and/or kinetics of thiocyanates compared to cyanides (for which conversion proceeds faster though the hydrolysis to cyanates).

### 3.4. Evolution of the Toxicity of the Solutions during the Photocatalytic Runs

To evaluate the impact of the photochemical treatments in the toxicity of the wastewaters, we used the *V. fischeri* luminescence inhibition test, as it is sensitive to the response of all the compounds present in the solution (cyanide species and intermediates formed). The method is based on the changes of the light emission in the luminescent bacterium under exposure to a toxic agent. As the light intensity is proportional to the metabolic activity of the bacterial population, a loss in the bioluminescence signal relates to the amount of toxic stress and corresponds to the toxicity of the solution (and vice versa) [31]. Furthermore, recent studies have shown that Microtox bioassay with *V. fischeri* bacterium is a good and practical method for screening the toxicity of wastewaters and aqueous solutions treated with advanced oxidation processes, as well organic and inorganic fractions of solid wastes [32,33,34].

The toxicity of a given compound to *V. fischeri* bacterium is usually referred as the mean effective concentration parameter (EC50), which is specific for each test system and each compound/mixture. EC50 represents the concentration of a compound that results in 50% reduction of the bacteria luminescence. The lower the EC50 value, the higher the toxicity. The corresponding EC50 values recorded for cyanide and thiocyanate are ca. 2 and 11 mg/L, respectively, and are in agreement with those reported in the literature [34]. However, EC50 descriptor cannot be estimated in multicomponent water samples with complex composition where the overall luminescence response of the bacteria encompass the contributions of all the compounds present in the water [21,31]. For this reason, we evaluated the toxicity of the as-received and treated wastewaters as the percentage of luminescence inhibition after exposure of the bacteria to different dilutions of the wastewaters in the range of 5–50% (dilution rates used in the Microtox test). The higher the inhibition percentage for a given dilution factor, the higher the toxicity.

**Figure 5:**
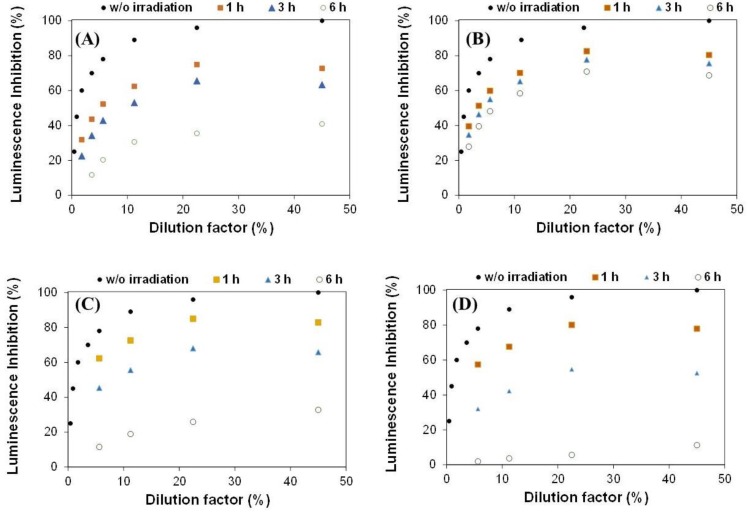
Luminescence inhibition of *V. fischeri* after exposure to cokemaking wastewater before (*w*/*o* irradiation) and after the photochemical treatments during 1, 3, and 6 h of irradiation using: (**A**) UVC lamp; (**B**) UV-Vis lamp; (**C**) SimSolar lamp; (**D**) SimSolar lamp and TiO_2_ as photocatalyst.

Figure 5 presents the inhibition of the luminescence signal obtained after 15 min exposure of the bacteria to the wastewaters before and after the photochemical treatment under the different illumination conditions. The pristine wastewater presented a moderate toxicity (i.e., 100% inhibition at 45% dilution). This is in agreement with the amount of cyanides and thiocyanates present in the initial wastewater (Table 1) and their corresponding EC50 values.

A decrease in the luminescence inhibition was observed after the exposure of the wastewater to light for all the irradiation sources, indicating that the treated waters are less toxic than the pristine one. This is mostly due to the transformation of cyanides and thiocyanates into less toxic intermediates (i.e., nitrates, ammonium) upon the photochemical treatment. Comparatively, lower values of toxicity (e.g., lower inhibition for a given dilution) were obtained after exposure to UVC and SimSolar lamps, compared to the UV-Vis lamp, particularly after long irradiation times. This indicates that there is a correlation between the intensity of the irradiation source and the decrease in the toxicity of the solution.

For further clarification, the comparison of the percentage of inhibition loss for a dilution of 45% for all the samples is shown in Figure S2. For all the illumination conditions, the toxicity decreased gradually with the irradiation time. This is in line with the evolution of the cyanide species (Figure 2) and the expected toxicity considering the residual concentrations of cyanide and thiocyanates in solution and their respective EC50 values. Irradiation during 1 h decreased the toxicity only slightly (i.e., 19–27% reduction of the luminescence inhibition) in the absence of a catalyst, thus the treated wastewater still presents a moderate toxicity. After 3–6 h of irradiation, the toxicity decreased more than 3 times after exposure to simulated solar light or to 254 nm. However, the effect was less pronounced for the illumination under the 200–600 lamp (e.g., 32% reduction of the luminescence inhibition after 6 h).

For the best performing conditions (ca. SimSolar and titania as photocatalyst), the reduction in the toxicity was very pronounced, particularly after 6 h of illumination (e.g., 88 % reduction of the luminescence inhibition for 45% dilution). Interestingly, there is still a non-negligible inhibition of the luminescence, despite the high conversion of thiocyanates and cyanides (Figure 1). This behavior cannot be attributed to the presence of the ionic intermediates (e.g., sulphates, ammonium, and nitrates), as their concentration did not modify the salinity of the bioassays assays—*V. Fischeri* is a marine bacterium and requires salinities between 2–8 w/vol. [35]—, which is kept constant by the osmotic adjustment of the solutions and their dilutions. Further analysis of the composition of the wastewater has revealed that this is attributed to trace concentration of quinones (EC50 of benzoquinone is ca. 0.025 mg/L), which are generated upon the photodegradation of phenolic compounds present in the pristine wastewater (Table 1).

## 4. Conclusions

We have investigated the simultaneous photooxidation of cyanides and thiocyanates from industrial wastewater with moderate concentrations of both species using various irradiation sources. The photochemical degradation of cyanides was more efficient and faster than that of thiocyanates, following a similar trend than that observed in synthetic solutions of the individual components. The photoconversion yield under simulated solar light was almost complete for cyanides and quite high for thiocyanates, in the absence of a catalyst and after 6 h of illumination. The oxidative degradation pathway is quite similar to that of non-radiative oxidation processes, involving the oxidation to cyanate, ammonia, nitrates, and nitrites. The nature of the predominant intermediate species depended on the illumination conditions. In the case of cyanide, direct photooxidation via reaction with the photogenerated holes and radical species is possible, rendering cyanate as intermediate, which is rapidly hydrolyzed into carbonates, ammonia, and nitrates. The exposure to a UVC lamp emitting at 254 nm resulted in a higher concentration of ammonia, compared to less energetic illumination sources. In the case of thiocyanate, the photochemical in homogeneous is very low due to the fast recombination reactions of the intermediate radical species. The overall yield is increased via charge transfer with electron scavengers (e.g., oxygen excess, catalyst) and when high energetic irradiation (e.g., 254 nm) is applied.

Regarding toxicity of the treated waters, the luminesce inhibition of *V. fischeri* bacteria decreased gradually with the irradiation time, indicating that the treated waters are less toxic than the pristine one. This is in agreement with the evolution of the cyanide species. Data showed that photochemical oxidation under UVC and simulated solar light was effective for lowering the toxicity of the pristine wastewater, indicating that there is a correlation between the intensity of the irradiation source and the decrease in the toxicity of the solution. These results indicate that simulated light can be effectively used to reduce the toxicity of industrial effluents, opening an interesting perspective for the optimization of cyanides detoxification systems based on natural light.

## Figures and Tables

**Table 1:** Main physicochemical parameters of the real wastewater from cokemaking factory.

Parameter	Value
pH	8.6
Conductivity (µS/cm, 20 °C)	1067
Suspended solids (mg/L)	39
Chemical oxygen demand (mg/L)	848
Total organic carbon (mg/L)	291
Phenols (mg/L)	4.2
Total hydrocarbons (mg/L)	2.2
Thiocyanates (mg/L)	226
Total cyanides (mg/L)	105

**Table 2:** Main characteristics of the light sources used in the photodegradation reactions.

	Wavelength (nm)	Irradiance (mW/cm^2^)	Remarks
**UVC**	254	26	Low pressure Hg lamp
**UV-Vis**	200–600	52	High pressure Hg lamp
**SimSolar**	200–800	190	Xe lamp, simulated solar light

**Table 3:** Molar yields of thiocyanate (SCN^−^) and cyanide (CN^−^) conversion to the intermediates detected in the solution (sulphates and N-containing species) after various illumination periods.

Lamp	Illumination Time	SCN^−^ to SO_4_^2−^	SCN^−^ and CN^−^ to N-species
UVC	1 h	0.99	0.81
UVC	3 h	0.99	0.97
UVC	6 h	0.99	0.99
UV-Vis	1 h	0.95	0.82
UV-Vis	3 h	0.99	0.87
UV-Vis	6 h	0.99	0.96
SimSolar	1 h	0.94	0.99
SimSolar	3 h	0.95	0.99
SimSolar	6 h	0.99	0.89
SimSolar + TiO_2_	1 h	0.98	0.92
SimSolar + TiO_2_	3 h	0.99	0.86
SimSolar + TiO_2_	6 h	0.99	0.98

## Supplementary Materials

The following are available online. Table S1. Main characteristics of commercial TiO_2_ used as photocatalyst. Figure S1. Emission spectra of the irradiation sources used in the photochemical assays. Figure S2. Bioluminescence inhibition loss at 45% dilution of the wastewater exposed to illumination under different conditions during 1,3, and 6 h.
