# Supplementary material for: Photochemical Degradation of Cyanides and Thiocyanates from an Industrial Wastewater
Source: Molecules. 2019 Apr 8;24(7):1373. doi: 10.3390/molecules24071373 (PMC6480199; doi:10.3390/molecules24071373)
Supplement: Supplementary file 1 [file molecules-24-01373-s001.pdf]

molecules

MDPI

Article

Photochemical degradation of cyanides and thiocyanates from an industrial wastewater

Juan José Viña Mediavilla[1], Begoña Fernandez Perez[1*], Maria C. Fernandez de Cordoba[2], Julia Ayala Espina[1], and Conchi O. Ania[2,3*]

[1] Escuela Técnica Superior de Ingeniería de Minas, Universidad de Oviedo, 33001 Oviedo, Spain; UO45242@uniovi.es; fernandezbegona@uniovi.es; jayala@uniovi.es
[2] CEMHTI, CNRS (UPR 3079), Université d'Orléans, 45071 Orléans, France; conchi.ania@cnrs-orleans.fr; maria.fdecordoba@cnrs-orleans.fr
[3] Instituto Nacional del Carbón (INCAR, CSIC), Apdo. 73, 33080 Oviedo, Spain; conchi.ania@cnrs-orleans.fr;

* Correspondence: conchi.ania@cnrs-orleans.fr; Tel.: +33-(0)-238-255-513; fernandezbegona@uniovi.es;

Supplementary Information File

Table S1. Main characteristics of commercial TiO_2 used as photocatalyst.

	$Area_{BET}$ ($m^2 g^{-1}$)	$V_{PORES}{}^{A}$ ($cm^3 g^{-1}$)	Optical Band gap (eV)	Surface pH
**TiO_2-P25**	53	0.083	3.2	6.4

A evaluated at relative pressure of 0.99 from N_2 adsorption isotherms at 77 K

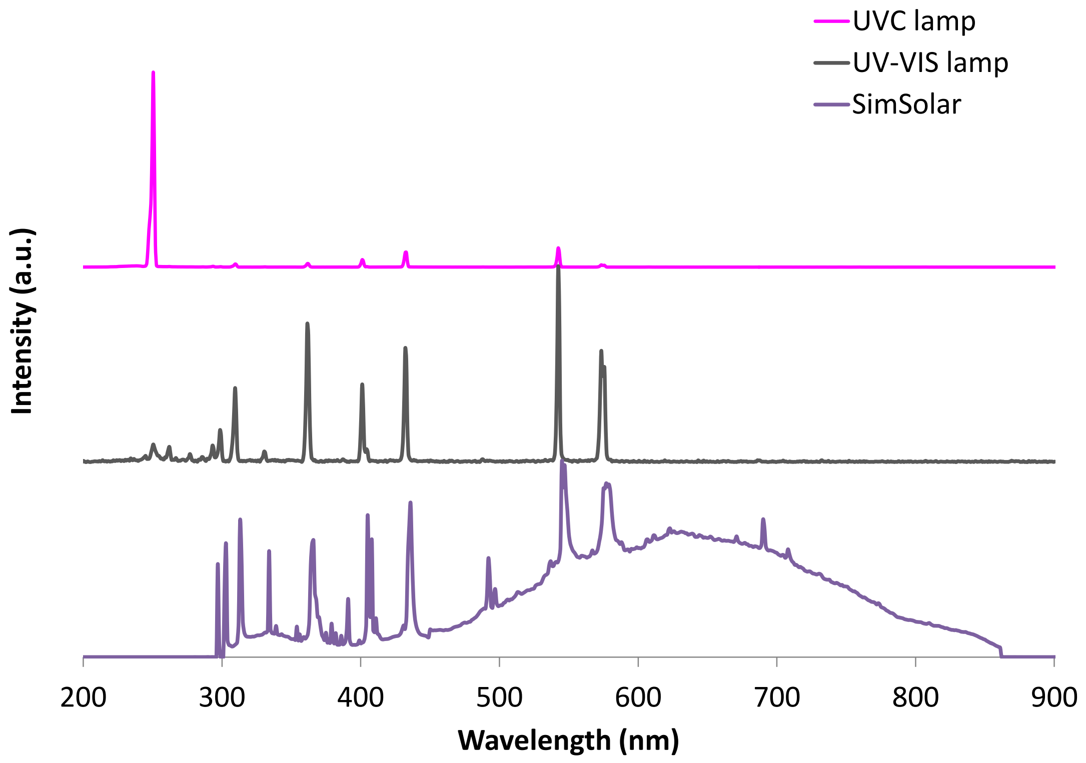

Figure S1. Emission spectra of the illumination sources.

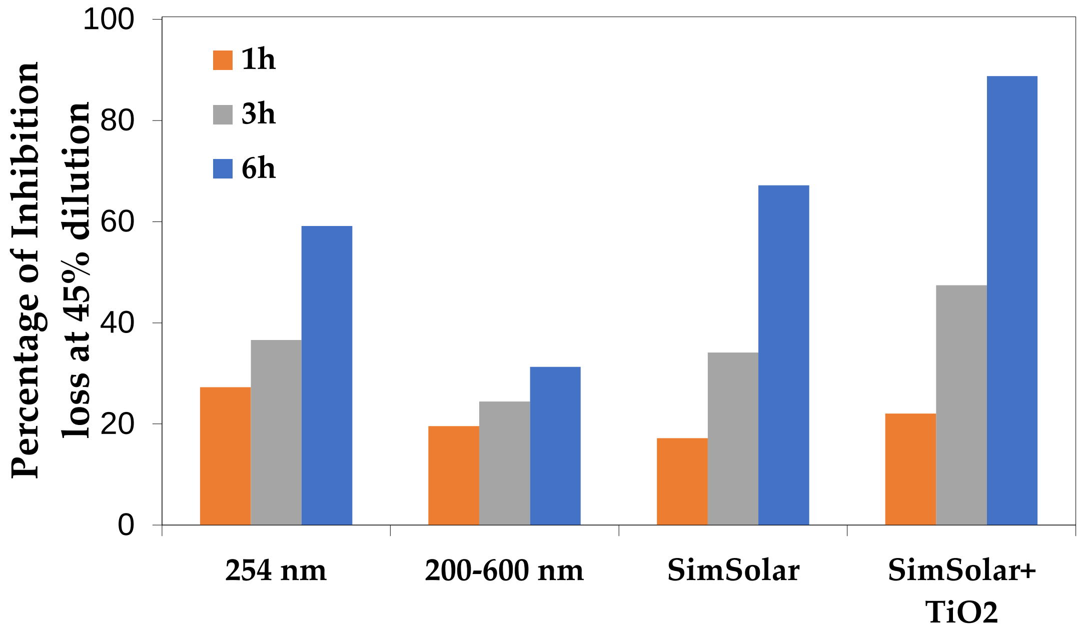

Figure S2. Bioluminescence inhibition loss at 45% dilution of the wastewater exposed to illumination under different conditions during 1,3 and 6 hours.
